# Thermal Management Technologies for Improving the Thermal Stability of Perovskite Solar Cells

**DOI:** 10.1007/s40820-025-02047-x

**Published:** 2026-01-13

**Authors:** Zhongquan Wan, Runmin Wei, Haibin Zhao, Wang Yu, Muhammad Azam, Junsheng Luo, Chunyang Jia

**Affiliations:** National Key Laboratory of Electronic Films and Integrated Devices, School of Integrated Circuit Science and Engineering, University of Electronic Science and Technology of China, Chengdu, 611731 People’s Republic of China

**Keywords:** Perovskite solar cells, Thermal degradation, Heat generation mechanism, Thermal stability, Thermal management technology

## Abstract

Perovskite solar cells (PSCs) have achieved excellent power conversion efficiencies; however, under direct sunlight, device temperatures can exceed ambient temperatures by more than 50 ℃, making thermal stability a critical challenge for commercialization. This review first summarizes the degradation mechanisms of PSCs induced by elevated temperatures, followed by a discussion of heat generation, with Joule heat identified as the primary contributor. Advanced thermal management strategies are then highlighted, including the use of high thermal conductivity materials, integration with thermoelectric devices, external radiative cooling layers, down-conversion approaches, and tandem structures. By systematically presenting these strategies, this review provides guidance for enhancing both the efficiency and thermal stability of PSCs, thereby supporting their pathway toward commercialization.

## Highlights

- Joule heating is the dominant cause of elevated device temperature in perovskite solar cells (PSCs) under operation, significantly degrades their long-term thermal stability.
- High temperatures degrade PSCs primarily through accelerated material decomposition and interfacial reactions, posing a major barrier to commercialization.
- Key thermal management strategies, such as integrating thermally conductive materials, radiative cooling layers, and tandem structures, effectively suppress heat accumulation and enhance device durability.

## Graphical abstract

**Figure:**
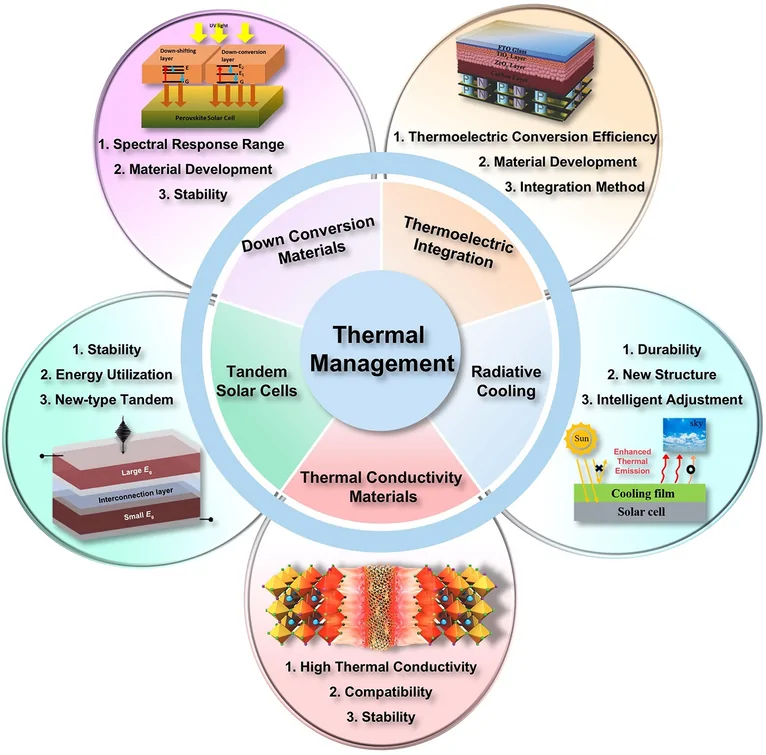

## Introduction

Energy is one of the most critical infrastructures in modern society, playing a vital role in industrial production and daily life. In recent years, as global climate change has intensified, governments worldwide have begun to increase their support for energy transitions, investing in and using renewable energy sources [1]. Among various renewable energy sources, solar energy is widely regarded as the most abundant, renewable, and environmentally friendly form on Earth. Research has shown that if we harness solar energy for photovoltaic (PV) power generation, utilizing just 0.1% of the solar radiation that reaches the Earth’s surface with an average power conversion efficiency (PCE) of 10%, the converted energy would be sufficient to meet our energy needs [2]. Therefore, converting solar energy into electricity is one of the most promising directions for achieving sustainable development [3, 4].

To date, silicon (Si) solar cells have dominated the PV market due to their high efficiency, mature manufacturing technology, and excellent stability. However, after achieving a PCE of 26.7% in 2017, the room for improvement in Si solar cells has become increasingly limited [5, 6]. In 2009, organic–inorganic perovskite solar cells (PSCs) first gained global scientific attention [7]. Perovskite materials have attracted widespread interest due to their high light-absorption coefficients, long carrier lifetimes, low cost, simple preparation processes, and scalability. Over the past decade, PSCs have become the most dazzling solar cells, showing immense commercial potential [8, 9]. Their PCEs have rapidly increased from 9.7 to over 26.7% [10]. However, alongside the rapid development of PSC technology, its poor thermal stability has gradually become evident, posing a significant challenge to large-scale commercialization [11].

Currently, researchers have made significant progress in improving the efficiency and stability of PSCs, with the main focus on strategies on such as interface engineering and bulk doping [12–17]. However, compared to these research areas, studies on thermal management of PSCs remain relatively scarce. The issue of thermal stability primarily stems from the thermal sensitivity of perovskite materials themselves. Under high-temperature conditions, ions within PSC tend to migrate, leading to material decomposition, interface degradation, and performance deterioration [11, 18]. This heat-induced degradation not only affects the long-term stability of PSCs but also severely limits the reliability in practical applications. Although interface engineering, bulk doping, and other methods can improve the thermal stability of PSCs to some extent, these strategies mainly target the chemical and physical properties of materials, enabling them to retain original characteristics at higher temperatures. However, they fail to effectively address the heat accumulation within PSCs [19].

Thermal management, which refers to effective strategies for regulating heat associated with devices by controlling temperature through heating or cooling (i.e., conduction, radiation, and convection) based on the specific requirements of object, has been widely applied in many fields such as light-emitting diodes, perovskite lasers, and Si solar cells to achieve longer lifespans [20, 21]. Many researches have shown that thermal management strategies can efficiently handle the waste heat generated within PSCs, thereby suppressing thermal degradation [19, 22–25]. However, there is a lack of systematic reviews summarizing and analyzing the current research status and future development directions in thermal management field of PSCs, which has motivated us to write this review, aiming to provide researchers with a comprehensive reference to advance the field of thermal management in PSCs.

In this review, we first delve into the thermal degradation mechanisms of PSCs under high–temperature conditions, focusing on analyzing the impact of high temperatures on the structural stability of perovskite materials, the properties of interfaces, and the overall performance of PSCs. Understanding the mechanisms of thermal degradation is the foundation for developing effective thermal management strategies. Next, we analyze the heat generation mechanisms within PSCs, which are divided into two parts: One part explores the internal heat sources, mainly including non-radiative recombination of photogenerated carriers and Joule heat during current transport; the other part focuses on external heat sources, such as increases in ambient temperature and solar irradiance. The combined effects of these heat sources lead to elevated device temperatures, accelerating the degradation process. A deep understanding of heat generation mechanisms helps guide the development of subsequent thermal management methods.

Finally, we concentrate on discussing thermal management strategies for PSCs, which are also the core content. We categorize the thermal management strategies into two major categories: The first category addresses the heat that has already been generated, taking measures to effectively dissipate or transfer it. Specific strategies include incorporating high thermal conductivity materials to improve heat dissipation, integrating external radiative cooling structures to release heat into the environment, and combining thermoelectric device to convert waste heat into electrical energy. The second category focuses on suppressing heat generation within the PSCs. It highlights the introduction of down-conversion (DC) materials inside PSCs and the construction of tandem solar cells (TSCs), where the energy that would otherwise be lost as heat is instead effectively harnessed for photoelectric conversion, thereby reducing heat generation at its source and enhancing the power conversion efficiency (PCE).

We systematically summarize the thermal degradation mechanisms, heat generation mechanisms, and existing thermal management strategies of PSCs, aiming to raise awareness of thermal stability issues and provide guidance for future research. We believe that with more in-depth research on thermal management, the thermal stability of PSCs will be significantly improved, creating more favorable conditions for commercialization. In future, as strategies such as thermal conductivity materials, radiative cooling structures, novel thermoelectric integration, DC materials, and TSCs continue to develop, the thermal management capabilities of PSCs will be continually enhanced, ultimately helping this emerging technology mature and become widespread.

## Thermal Degradation Mechanisms of PSCs

Solar panels are often installed outdoors and exposed to direct sunlight, especially under hot weather conditions, causing the operating temperature of PSCs to sometimes exceed 85 ℃. To enter the commercial market, PSCs must exhibit long-term stability at a temperature of at least 85 ℃ [26]. Therefore, we typically test PSC at 85 ℃ to assess issues such as thermal degradation or performance decline, ensuring the reliability and durability of PSCs. At present, different degradation mechanisms of perovskite materials under the influence of temperature have been proposed. Taking commonly used MAPbI_3_ with ABX_3_ structure as an example, the degradation reactions of MAPbI_3_ and MAI are as follows [27]:

1 $${\mathrm{MAPbI}}_{{3}} \to {\mathrm{NH}}_{{3}} + {\mathrm{CH}}_{{3}} {\mathrm{I}} + {\mathrm{PbI}}_{{2}}$$

2 $${\mathrm{MAI}} \to {\mathrm{NH}}_{{3}} + {\mathrm{CH}}_{{3}} {\mathrm{I}}$$

**Fig. 1:**
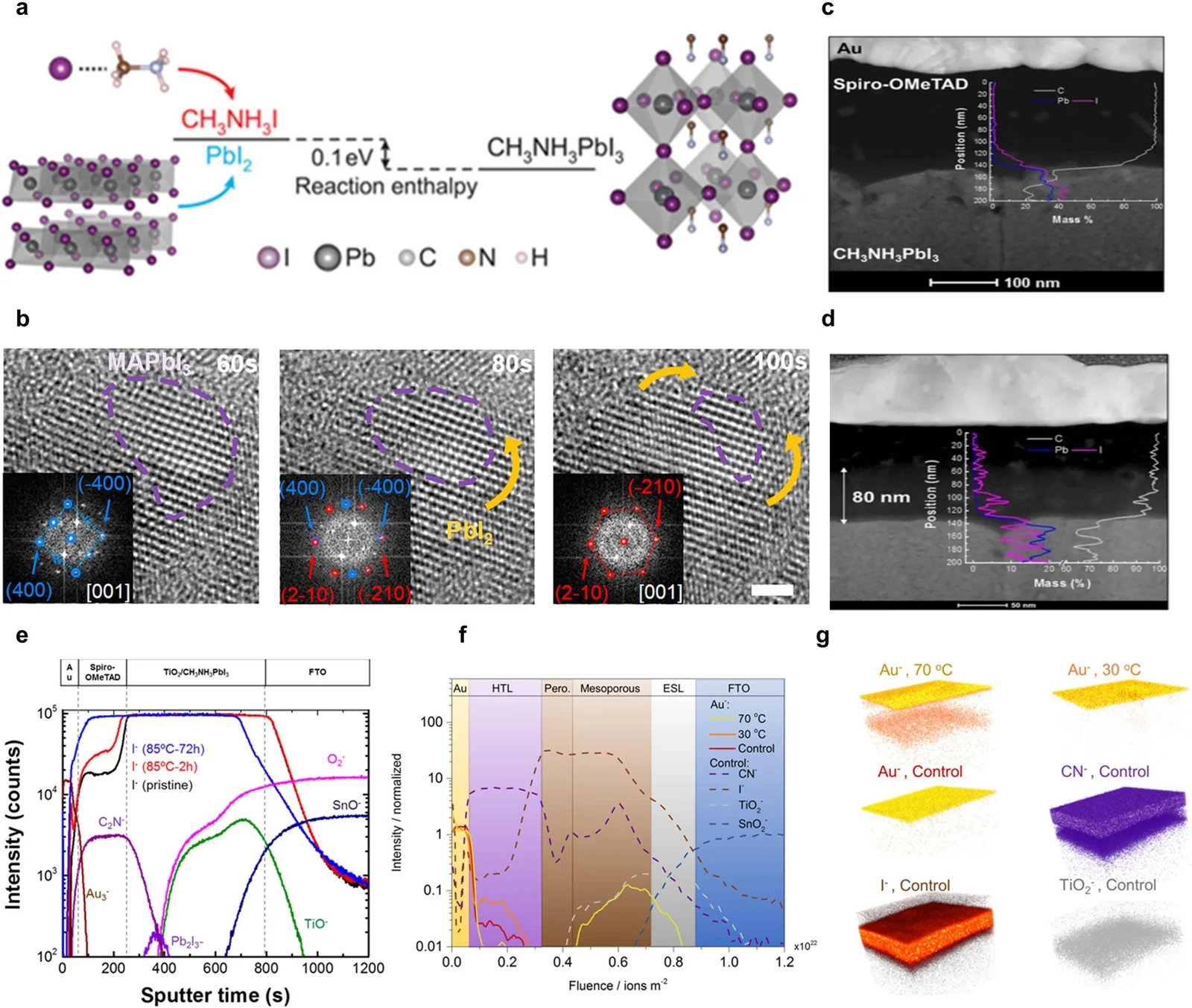
**a** Schematic diagram of tetragonal MAPbI_3_ [29]. **b** Degradation process of an individual MAPbI_3_ grain [30]. HAADF STEM images of the MAPbI_3_/Spiro-OMeTAD interface: **c** before and **d** after heating at 85 ℃ for 2 h. Inset shows EDX line maps for lead and iodine [34]. **e** TOF–SIMS depth profiles of the aged PSC devices [34]. **f** TOF–SIMS depth profiles of the concentration of selected species across the control device. The profile of Au^–^ is compared to that of the devices aged at 30 and 70 °C [35]. **g** Reconstructed elemental 3D maps for the ions traced in the depth profile. The *xy* dimensions of the analyzed area are 10 × 10 μm [35]

Equation 1 depicts that MAPbI_3_ first decomposes into PbI_2_ upon long-term heat treatment in a range of 85–200 °C [28]. The PbI_2_ formed is dispersed in perovskite layer of PSC, acting as defect centers that exacerbate electron–hole recombination. Figure 1a illustrates the schematic diagram of low formation energy (0.1 eV) synthesis of tetragonal MAPbI_3_, implying that the reversible reaction shifts to left at elevated temperature, decomposing into PbI_2_ and MAI [29]. Based on differential scanning calorimetry and quadrupole mass spectrometry measurements, Zhang et al. showed that MAPbI_3_ and MAI eventually decompose into PbI_2_, NH_3_ gas, and a toxic CH_3_I solution, leading to a rapid decrease in PCE during this process [11]. Fan et al. studied the degradation mechanism of highly crystalline MAPbI_3_ under thermal stimulation using in situ high-resolution transmission electron microscopy (HRTEM) (Fig. 1b). After heating at 85 °C for 100 s, nearly 75% of the original tetragonal perovskite phase reverted to the orthorhombic PbI_2_ phase [30]. Kumar et al. employed low-frequency noise measurements to investigate the degradation mechanisms in PSCs. Their research indicated that the significant decline in PSCs performance at elevated temperatures was primarily due to the increased recombination of charge carriers, which affected the open-circuit voltage (*V*_oc_). Furthermore, the aging studies conducted at various temperatures demonstrated that PSCs exhibited high stability at temperatures below 70 °C, but underwent severe irreversible degradation at higher temperatures [31].

Apart from the perovskite layer, other functional layers also undergo changes at high temperatures. Malinauskas et al. presented that the amorphous Spiro-OMeTAD, widely used as a hole transport material (HTM), was unstable at temperatures above 100 °C due to crystallization [32]. The large crystals formed in the amorphous Spiro-OMeTAD lead to hole traps, which then affected the charge transport. Although hole dopants are very important to increase the conductivity of Spiro-OMeTAD, they also play a vital role in the thermal degradation of PSCs [33]. Dopants employed to increase the hole transport and reduce the glass transition temperature of Spiro-OMeTAD tend to result in crystallization under high temperature, which leads to the poor performance of PSCs. Kim et al. analyzed iodine ions at MAPbI_3_/Spiro-OMeTAD interface using high-angle annular dark-field (HAADF) scanning transmission electron microscopy (STEM) and energy-dispersive X-ray spectroscopy (EDX), and found that the initial distribution of iodine ions was within a depth of less than 20 nm from the interface. After heating at 85 °C for two hours, time-of-flight secondary ion mass spectrometry (TOF–SIMS) confirmed iodine ions migrated to a depth of approximately 80 nm into Spiro-OMeTAD (Fig. 1c–e). Additionally, X-ray photoelectron spectroscopy (XPS) results showed that not only iodine ions, but also methylammonium ions diffused from perovskite layer into Spiro-OMeTAD, altering the chemical property of Spiro-OMeTAD and severely affecting the performance of hole transport layer (HTL) [34]. Domanski et al. observed significant gold migration from the electrode through the HTL to the perovskite layer during PSC aging at 70 °C, as revealed by TOF–SIMS analysis (Fig. 1f, g). The presence of gold in the perovskite layer not only forms short-circuit pathways leading to a sharp decline in *V*_oc_ but also creates deep trap states that enhance non-radiative recombination, reducing *J*_sc_; it increases series resistance and decreases shunt resistance, jointly lowering FF, and accelerates irreversible PCE decay under prolonged thermal stress, worsening long-term stability [35].

## Heat Generation Mechanisms in PSCs

The temperature rise in PSCs primarily stems from two sources: external environmental thermal effects and intrinsic thermal effects generated during operation. Environmental thermal effects include factors such as solar radiation, ambient temperature, wind speed, and humidity. Intrinsic thermal effects involve thermalization heat, non-radiative recombination heat, Peltier heat, and Joule heat [36]. Reducing the generation of these two types of heat is a primary goal for optimizing the overall thermal performance of PSCs. Therefore, a deep understanding of the heat generation mechanisms in PSCs is crucial for researchers.

**Fig. 2:**
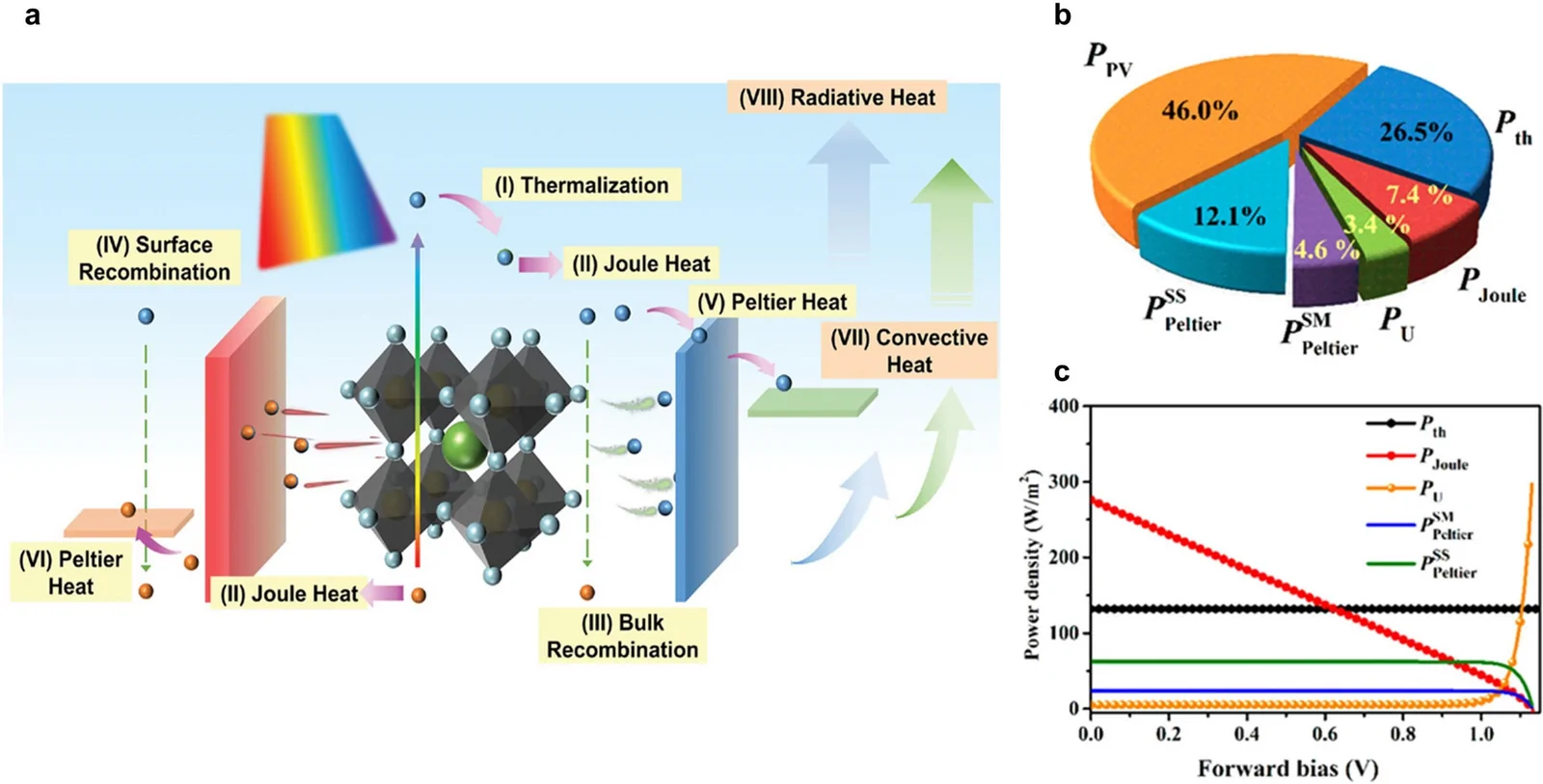
**a** Energy conversion processes of a PSC concerning: (I) thermalization heat, (II) Joule heat, (III) bulk recombination heat, (IV) surface recombination heat, Peltier heat at (V) heterojunction interface and (VI) metal/semiconductor interface. Heat dissipation processes include VII) convective heat and (VIII) radiative heat [36]. **b** Pie chart of microscopic energy conversion components at MPP [39]. **c** Simulated power density of heat conversion as a function of forward bias [39]

Figure 2a illustrates the microscopic energy conversion processes between photons, charge carriers, and phonons in PSCs. From the perspective of recombination sources, the thermal effects can be categorized into six types: (1) Thermalization heat generated by energy relaxation, where photo-excited electrons (holes) with excess potential energy (*hv*-*E*_g_) beyond the bandgap release this energy within picoseconds, returning the electrons (holes) to the conduction (valence) band edge; (2) Joule heat generated as charge carriers pass through the depletion region under the influence of the built-in electric field [37]; (3) Bulk recombination heat caused by Shockley–Read–Hall (SRH) and Auger recombination; (4) Surface recombination heat generated when carriers are captured by surface defects; (5) Peltier heat at heterojunction interface due to the energy band offset; (6) Peltier heat at the semiconductor/metal interface, where the transport carriers have to flow from conduction/valence band of semiconductor region to the quasi-Fermi level before being collected by the respective electrodes [38]. Additionally, there are macroscopic heat exchange processes, including thermal convection and thermal radiation. It is also worth noting that these heat generation mechanisms are not entirely independent but often interact within the device. For instance, excessive Joule heat can accelerate non-radiative recombination by increasing carrier scattering and defect activation, thereby amplifying recombination heat. Similarly, thermalization and recombination processes may locally modify carrier distributions, indirectly affecting current flow and thus Joule heat. Moreover, Peltier effects at interfaces can either mitigate or enhance local heating depending on current direction and contact properties. These coupling effects highlight the complex thermal dynamics in PSCs, emphasizing the need for comprehensive thermal management strategies that consider both individual and synergistic heat generation processes.

An et al. developed an opto-electro-thermal (OET) model to elucidate the mechanisms of heat generation, dissipation, and regulation in PSCs [39]. Through detailed calculations, they provided an energy conversion diagram for PSCs operating at the maximum power point (MPP). The results showed that photovoltaic conversion (*P*_pv_), thermalization heat loss (*P*_th_), Joule heat (*P*_Joule_), heterojunction interface Peltier heat (*P*_Peltier_^SS^), semiconductor–metal interface Peltier heat (*P*_Peltier_^SM^), and total recombination heat (*P*_U_) accounted for 46%, 26.5%, 12.1%, 7.4%, 4.6%, and 3.4% of incident energy, respectively. This result indicates that only 46% of the incident energy is converted into electricity, and nearly 54% is converted into heat, with the thermal impact of thermalization heat on PSCs being the greatest (Fig. 2b).

Given that the heat sources arise from different mechanisms, An et al. also simulated the relationship between thermal conversion power density and forward bias voltage (Fig. 2c) and proposed corresponding thermal management strategies: (1) Thermalization heat is solely determined by material bandgaps and is independent of applied bias. Constructing gradient bandgap materials or multi-junction configurations is an effective strategy to reduce thermalization heat generation [40–42]; (2) Increasing forward bias reduces the built-in electric field in PSCs, thereby lowering carrier separation capability and increasing recombination current, gradually leading to increased recombination heat. Optimizing carrier transport dynamics can significantly reduce recombination heat [43–45]; (3) Joule heat is typically regarded as an inherent self-heating effect in PSCs, making it difficult to modify [46]; (4) Peltier heat (*P*^SS^_Peltier_ and *P*^SM^_Peltier_) depends on current density in PSCs and varies with current density. Effective suppression of Peltier heat can be achieved by reducing band offsets at heterojunctions and semiconductor–metal interfaces.

In summary, the heat sources in PSCs primarily include external environmental factors and intrinsic thermal effects generated during operation. These heat sources not only affect the efficiency of PSCs but also pose significant threats to long-term stability. By deeply analyzing the mechanisms of thermalization heat, recombination heat, Joule heat, and Peltier heat, we can better understand their impacts on the performance of PSCs and propose targeted thermal management strategies. In the following section, we will systematically introduce and discuss various existing thermal management strategies, aiming to address the issue of poor thermal stability in PSCs and pave the way for the practical applications.

## Strategies for Heat Dissipation within PSCs

**Fig. 3:**
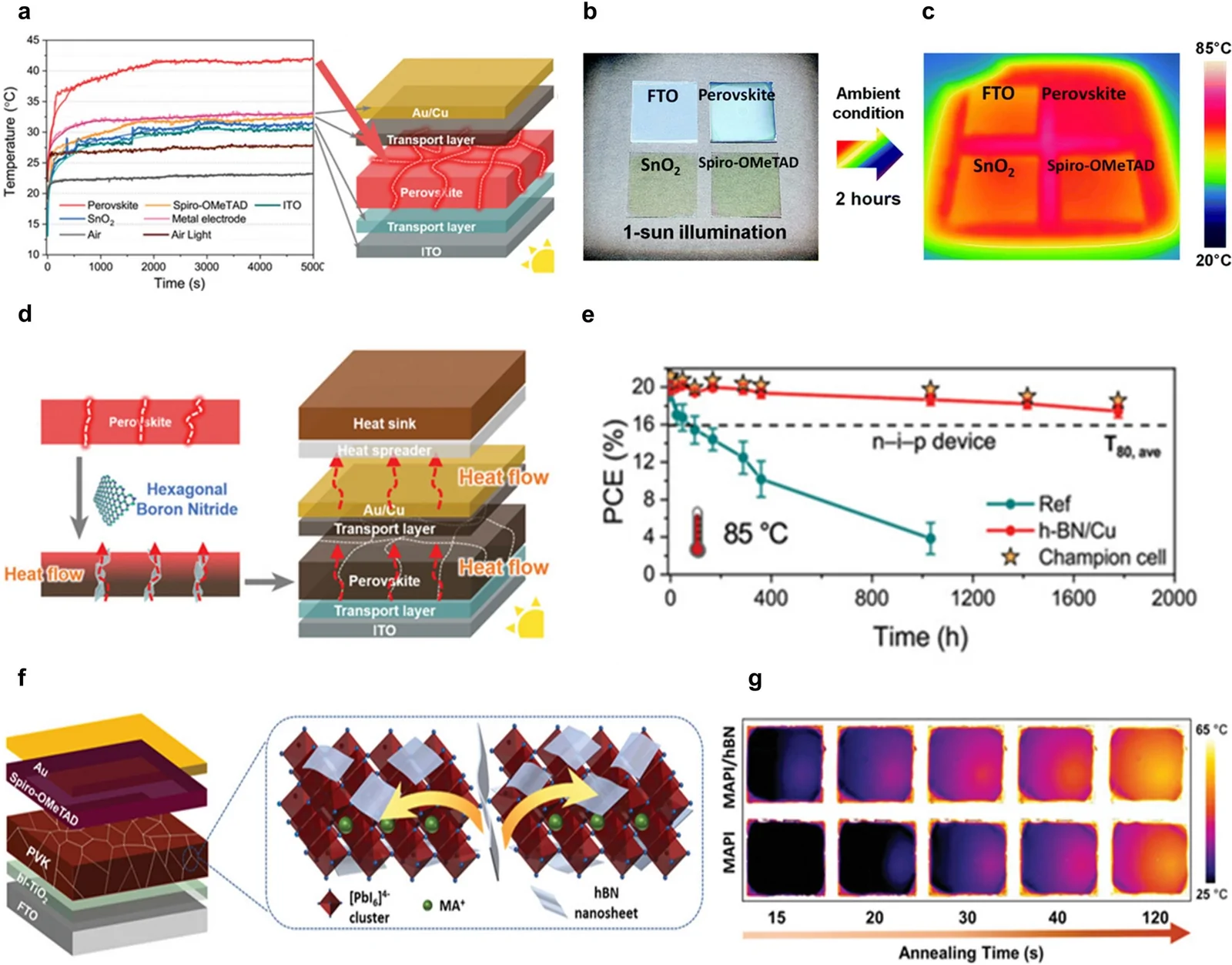
**a** Real-time temperature tracking for each functional layers in PSCs under continuous illumination (AM 1.5G, 100 mW cm^−2^) [19]. IR thermal images of each functional layers prepared on the glass substrate **b** before and **c** after 1 sun illumination [49]. **d** Schematic diagram of PSCs with improved heat transfer by using h-BN [19]. **e** Normalized PCE of PSCs aging under different conditions [19]. **f** Schematic diagram of PSCs with h-BN [50]. **g** Annealing process of perovskite layers with h-BN nanosheets over time [50]

PSCs have functional layers with extremely low thermal conductivity [23, 47, 48]. It means that any heat generated during operation accumulates within the PSCs, leading to a continuous increase in operating temperature, which significantly impacts performance. Yang et al. monitored the surface temperatures of PSC’s various functional layers under standard sunlight exposure [19]. The temperature of perovskite layer was approximately 20 °C higher than the ambient air temperature, while the temperatures of other functional layers were about 10 °C higher (Fig. 3a). Choi et al. used infrared cameras to observe the thermal response of functional layers under standard sunlight exposure for 2 h, revealing significant temperature differences among the layers [49] (Fig. 3b, c). The results had shown that perovskite layer reached a maximum temperature of 75 °C due to its excellent light-absorption characteristic. In real world with intense and prolonged sunlight exposure, temperatures could even exceed 75 °C. Elevated temperature directly leads to performance degradation in PSCs. In this section, we review the existing thermal management strategies that effectively address the heat accumulation in PSCs, such as improving thermal conductivity of functional layers, integrating thermoelectric device, and radiative cooling strategy. Furthermore, the key parameters of each strategy are summarized in Table 1 at the end of this section.

**Table 1:** Optoelectronic performance and stability of PSCs with different thermal management strategries to treat the heat within PSCs

Thermal management strategies	*V*_oc_ (V)	*J*_sc_ (mA cm^−2^)	FF (%)	PCE (%)	Stability/thermal performance of device	Ref
h-BN inside perovskite layer and radiator fin outside of PSC	1.120	25.20	80.40	22.77	Retained 96 and 92% of its initial PCE after 1704 h of 85 °C thermal aging and 2164 h of MPP tracking	[19]
2D h-BN nanosheets inside perovskite layer	1.095	23.81	76.00	19.80	Retained ≈ 90% of its initial PCE after 500 h ambient air storage	[50]
Multi-walled carbon nanotubes inside perovskite layer	–	–	–	23.05	Retained 89% of its initial PCE after 1300 h of 85 °C thermal aging	[51]
1-Butyl-3-methylimidazole dibutyl phosphate (BMDP) inside perovskite layer	1.136	24.70	82.85	23.25	Retained about 90% of its initial PCE, whether at 85 °C for 1080 h, or in air (RH = 30 ± 5%, 25 ℃) for 1440 h	[52]
Al_2_O_3_ NPs inside HTL	1.190	22.70	78.80	21.20	Retained 91% of its initial PCE after 31 days at 85 °C and RH = 85%	[49]
SiO_2_ particles at the perovskite/HTL interface	1.130	24.18	81.84	22.29	Retained 91% and 95% of its initial PCE after thermal aging at 85 °C for 1126 h and SPO operation for 1235 h in a N_2_ atmosphere, respectively	[23]
Zeolite inside HTL	1.160	25.21	80.10	23.42	The Spiro-OMeTAD based and PTAA based device maintained 61 and 92% of its initial PCE after 720 h of thermal aging at 85 °C under N_2_ atmosphere	[56]
A small amount of polystyrene (PS) inside PC_71_BM electron transport layer	1.070	22.90	75.20	18.34	Retained 64% of its initial PCE after 100 h at 65 °C	[25]
Integration of carbon counter electrode based PSC with TEG	1.870	19.80	60.00	22.20	When the temperature increased from 30 to 100 ℃, the *V*_oc_ of the integrated device increased from 1.87 to 2.59 V because of the accumulated voltage generated by the TEG, compensating the thermal degradation of PSC	[73]
Integration of hole-conductor-free PSC based on TiO_2_/ZrO_2_/carbon structure and TEG	1.290	22.80	69.00	20.30	–	[64]
Large-area (16 cm^2^) perovskite solar module –TEG-integrated device	6.670	3.06	58.60	11.96	Retained 85% of its initial PCE after 400 h under continuous illumination	[62]
All-inorganic, full-spectral concentrator CsPbBr_3_/Bi_2_Te_3_-integrated solar cells	2.114	38.25	77.03	12.46	Retained over 90% of its initial PCE after 150 h irradiation under 5 suns	[76]
Membrane-free redox flow cell based on thermally regenerative electrochemical cycle for concurrent electricity storage, cooling and waste heat harnessing of PSCs	–	–	–	–	The temperature of PSC could be reduced from 60 °C to below 35 ℃	[77]
Pyramid-structured PDMS radiative cooling layer for PSCs	–	–	–	–	The pyramid-structured PDMS layer can significantly lower the temperature of PSC by 12 °C	[83]
Radiative cooling layer for PSCs	–	–	–	–	Using such a photonic radiative cooler could lower the temperature of PSCs by more than 10 °C and increase the PCE by 0.45%	[81]
Submicron organic − inorganic hybrid radiative cooler for PSCs	0.941	17.64	75.30	12.50	Integrating photonic cooling in PSCs can result in temperature reductions of up to ∼ 17 K	[84]
A grating-textured PDMS photonic structure for PSCs	–	–	–	–	Numerical analysis showed that the radiative cooler can ideally achieve a temperature reduction of 11.47 K for encapsulated PSCs	[85]

### Innovative Thermal Conductivity Materials in Thermal Management of PSCs

The ultra-low thermal conductivity (around 0.2 W m^−1^ K^−1^) of perovskite layer severely affects heat dissipation within PSCs [23, 47, 48]. Yang et al. first proposed introducing hexagonal boron nitride (h-BN) with high thermal conductivity into perovskite layer to create thermal pathways and accelerate heat dissipation internally [19] (Fig. 3d). It was verified that h-BN mainly distributes at the grain boundaries of perovskite layer, where heat tends to accumulate. Additionally, h-BN possessed properties such as high spatial hindrance and weak electron affinity, which does not disrupt the perovskite crystal lattice but coexisted with it complementarily in the perovskite layer. PSCs fabricated using this method, both n-i-p and p-i-n structures, exhibited exceptionally high *T*_80_ (time required for efficiency to decrease to 80% of the initial PCE) lifetimes under aging at 85 °C. It was noteworthy in their study that besides enhancing internal heat dissipation, additional Cu heat sink was installed at the top electrode, resulting in n-i-p-structured PSC based on h-BN/Cu heat sink retaining 88% and 93% of its initial PCE after thermal aging for 1776 h and MPP tracking for 2451 h under 85 °C, respectively. Simultaneously, p-i-n-structured PSC based on h-BN/Cu heat sink maintained 96% and 92% of its initial PCE after thermal aging for 1704 h and MPP tracking for 2164 h under 85 °C (Fig. 3e).

Yin et al. also incorporated 2D h-BN nanosheets with high thermal conductivity as additives into perovskite layer [50]. They found that h-BN nanosheets enhanced the heat transfer of perovskite layer, leading to faster and more uniformly distributed heating during the annealing process (Fig. 3f, g), which promoted phase transitions and grain growth within perovskite layer. Moreover, although h-BN is electrically insulating, its effect on charge transport is minimal because it localizes primarily at grain boundaries rather than within the conductive perovskite grains. In this position, h-BN can passivate defects and suppress ion migration without obstructing carrier pathways. Consequently, the improvements in *V*_oc_ and FF indicate that its insulating nature does not compromise device performance. The combined benefits of enhanced heat dissipation and interface passivation contribute to increased efficiency and operational stability of perovskite device.

**Fig. 4:**
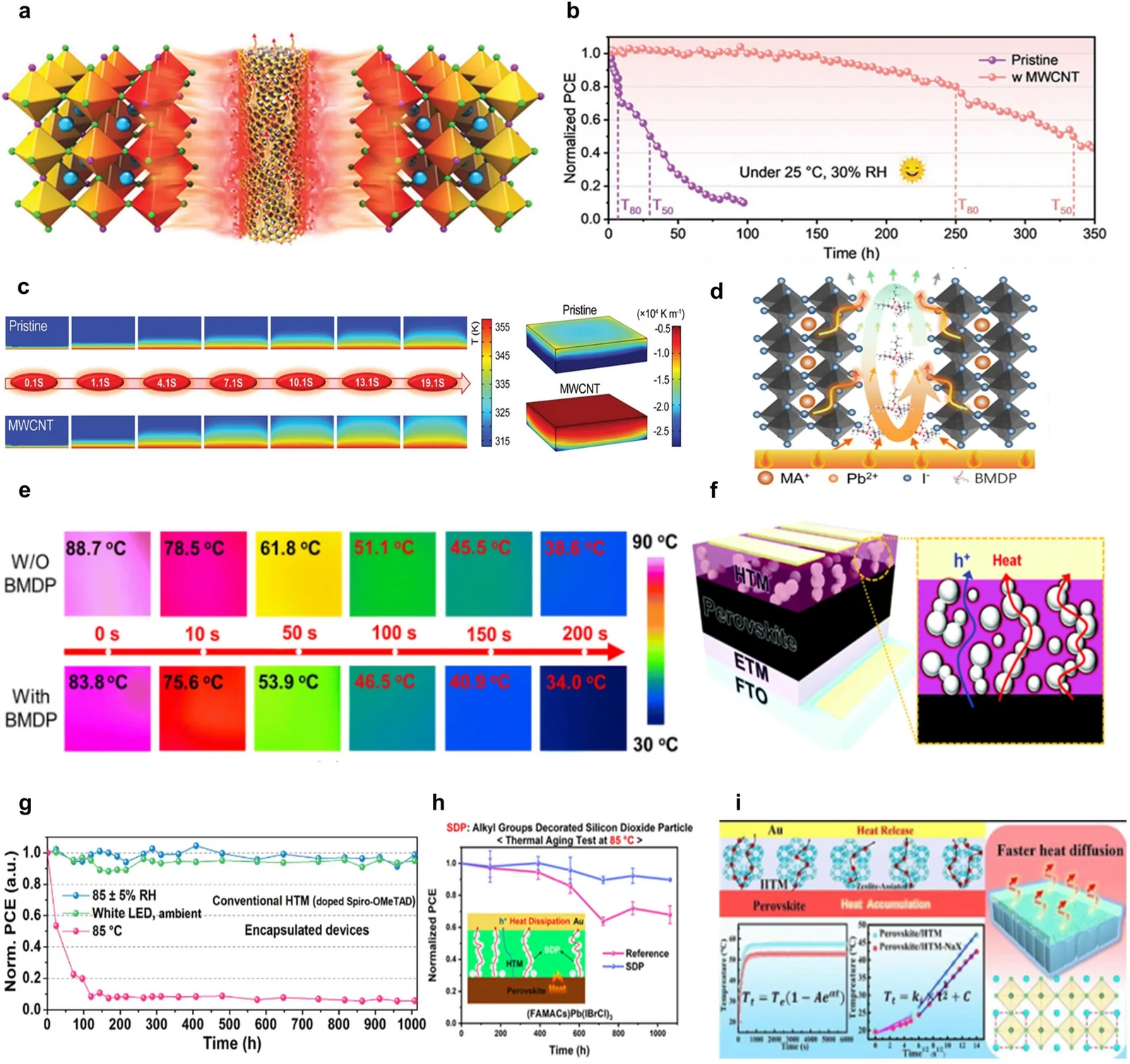
**a** Schematic diagram of heat transfer in perovskite modified by MWCNTs [51]. **b** SPO stability of PSCs operating at bias near maximum power output point at 25 °C and 30% RH with encapsulation [51]. **c** Thermal diffusion coefficients under 85 ℃ of various perovskite layers [51]. **d** Schematic diagram of thermal management based on BMDP [53]. **e** IR thermal images of ITO/PTAA/MAPbI_3_ and ITO/PTAA/MAPbI_3_/BMDP films under a cooling test [53]. **f** Schematic diagram of PSC using Al_2_O_3_ NPs as thermal conductivity materials [49]. **g** Long-term stability of encapsulated devices based on doped Spiro-OMeTAD at various conditions [49]. **h** Long-term thermal stability of unencapsulated PSC using SiO_2_ NPs as the interfacial material [23]. **i** A universal zeolite-assisted heat conduction strategy was employed to maintain the high efficiency and stability of overheated PSC [24]

Li et al. improved the thermal conductivity of perovskite layer using multi-walled carbon nanotubes (MWCNTs) where the outer layer was functionalized to enhance interaction with perovskite, while the inner layer maintained excellent thermal conductivity [51] (Fig. 4a). They measured the thermal diffusivity and thermal conductivity of modified perovskite using laser flash and Hot-Disk techniques, respectively, demonstrating significant enhancement in thermal performance. Ultimately, the MWCNT-modified devices demonstrate outstanding stability (Fig. 4b). Innovatively, they applied finite element analysis based on COMSOL software to model the internal thermal conduction during annealing of perovskite layer, explaining microscopic changes not characterized by existing testing methods (Fig. 4c). The simulation results showed that MWCNTs can improve heat transfer and reduce longitudinal temperature gradients during annealing, ensuring uniform annealing for the formation of dense, large-grain perovskite layers.

Ionic liquids are commonly used as heat transfer fluids in thermal conductivity materials, and they also can act as passivating agents in PSCs [52]. Zhang et al. first discovered an ionic liquid 1-butyl-3-methylimidazolium dibutylphosphate (BMDP) with high electrical conductivity and good thermal stability, which was widely used as a green solvent in chemical reactions and flame-retardant additives [53]. Its low viscosity and high fluidity allowed easy filling into grain boundaries of perovskite, forming liquid domains in grain boundaries as excellent heat transfer media [54]. Furthermore, the negatively charged imidazole group and phosphate group can interact with perovskite via Lewis acid–base reaction and ion interaction, and may act as an efficient passivator in grain boundaries and the surface of perovskite layers to reduce the defects [55] (Fig. 4d). The excellent thermal stability and performance of PSC based on BMDP achieved the *T*_90_ over 970 h at 85 °C and the high PCE of 21.98%, which was much better than that of control PSC (Fig. 4e). This work presented a novel multifunctional thermal management strategy for PSCs.

While the low thermal conductivity of perovskite layers is a critical factor leading to heat accumulation inside PSCs, effective thermal pathways constructed in other functional layers can also accelerate internal heat conduction. Choi et al. first introduced metal oxide Al_2_O_3_ nanoparticles (NPs) into HTL utilizing their thermal conductivity and porous scaffold structure to dissipate heat accumulated within PSCs [49] (Fig. 4f). Unencapsulated PSC modification with Al_2_O_3_ NPs retained 91% of initial PCE after 31 days under harsh environments (85 °C, RH = 85%) (Fig. 4g). This method for improving the thermal conductivity of HTL was proposed for the first time by them. Subsequently, Pei et al. introduced thermal conductivity SiO_2_ NPs into HTL [23]. SiO_2_ not only enhanced the thermal performance of HTL but also coordinated with under-coordinated Pb^2+^ as a passivation agent for the perovskite surface. Ultimately, the unencapsulated PSC based on SiO_2_ retained 91 and 95% of its initial PCE after 1126 h of thermal aging at 85 °C and 1235 h of operation in a N_2_ atmosphere, respectively (Fig. 4h).

Zeolites are a class of structurally ordered porous inorganic aluminosilicates known for high chemical and thermal stability [24, 56]. Zeolites with high framework density are typically composed of alternating TO_4_ tetrahedra connected by shared vertex O atoms, exhibiting potential thermal conductivity along with long-range organized frameworks [57, 58]. Alternating TO_4_ structural units provide zeolites with efficient thermal diffusion pathways, facilitating the heat transfer through T–O chemical bonds and diffusion within the three-dimensional TO_4_ tetrahedral frameworks. Additionally, lattice pores with negative charges can effectively transport cations [59, 60]. Wang et al. utilized these properties of zeolites by incorporating them into HTL, enabling rapid cooling of the entire PSC and promoting charge transfer [61]. Zeolite-modified PSC exhibited excellent stability after heating at 85 °C under N_2_ conditions for 1000 h (Fig. 4i). Currently, there is limited research on improving the heat transfer performances of electron transport layers. Zhang et al. blended PC_71_BM with a small amount of the commonly used insulating polymer polystyrene (PS) to improve the thermal conductivity of PC_71_BM [25].

In summary, the integration of high thermal conductivity materials into PSCs has emerged as a pivotal thermal management strategy, significantly enhancing heat dissipation and operational stability. However, the efficacy of these materials is contingent upon their intrinsic properties and integration methodology. Materials such as h-BN, SnO_2_, and Al_2_O_3_ exhibit high thermal conductivity (20–30 W m^−1^ K^−1^) but are electrically insulating, which can compromise charge transport if not optimally incorporated. In contrast, CNTs offer exceptional thermal and electrical conductivity (~ 600–3000 W m^−1^ K^−1^), yet face challenges with aggregation and interfacial defects. Ionic liquids (e.g., BMDP) provide a multifunctional solution, combining moderate thermal conductivity (~ 0.76 W m^−1^ K^−1^) with defect passivation and ion migration suppression, though their low thermal conductivity requires complementary strategies. Zeolites leverage their ordered porous structure to facilitate heat transfer and defect passivation, but their complex synthesis may limit scalability.

Future advancements should focus on optimizing the compatibility and distribution of these materials within functional layers to minimize adverse effects on electrical performance. Combining multiple strategies (e.g., CNTs with ionic liquids) and employing advanced manufacturing techniques could synergistically enhance both thermal and electrical performance, paving the way for commercially viable PSCs.

### Thermoelectric Integration for Heat Management of PSCs

**Fig. 5:**
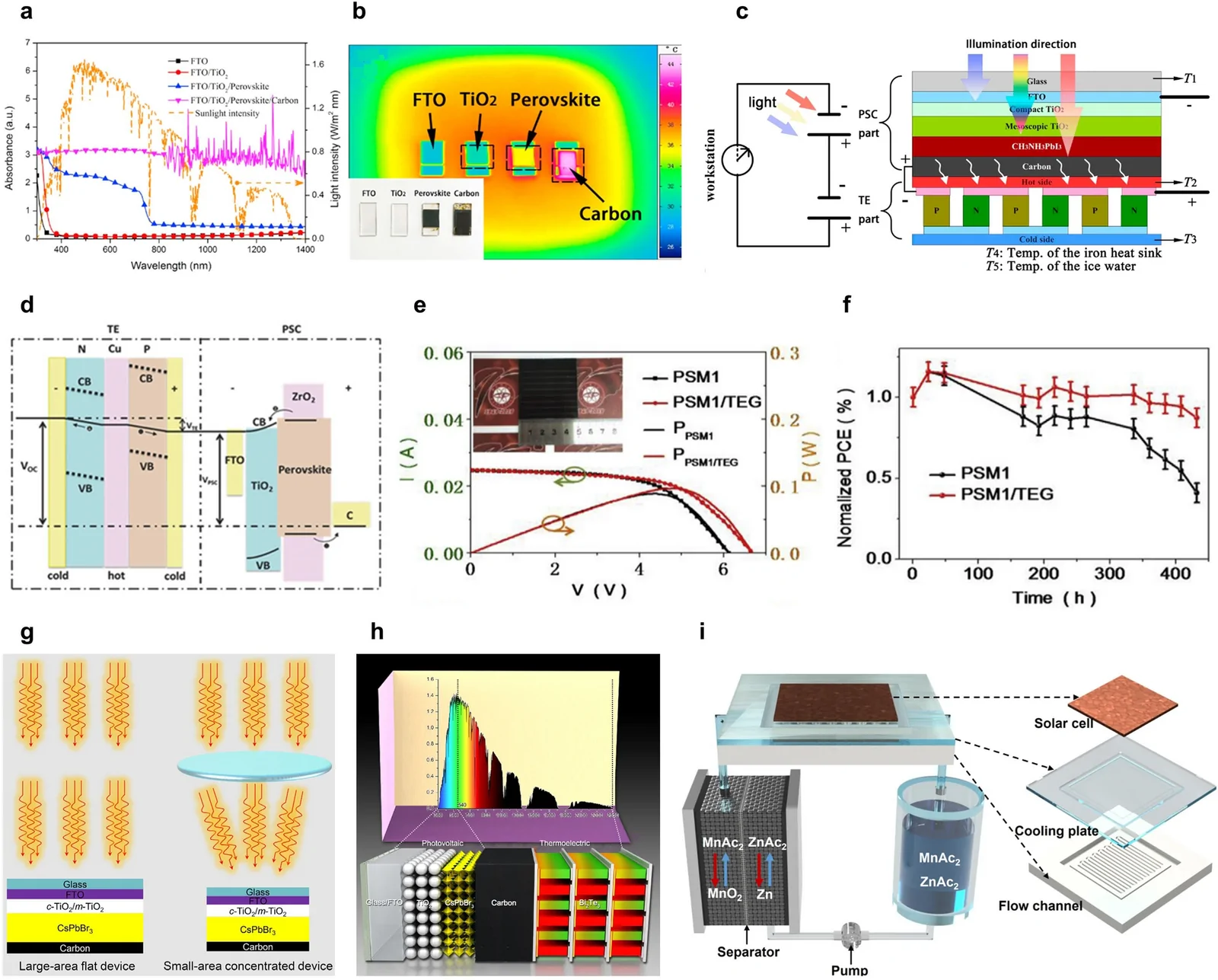
**a** UV–Vis–NIR spectra of FTO, FTO/TiO_2_, FTO/TiO_2_/perovskite, and FTO/TiO_2_/perovskite/carbon layers [73]. **b** Thermal NIR images and photographic images (in the inset) of the FTO, FTO/TiO_2_, FTO/TiO_2_/perovskite, and FTO/TiO_2_/perovskite/carbon layers under sunlight (AM 1.5G, 100 mW cm^−2^) [73]. **c** Schematic diagram of PSC–TEG-integrated device [73]. **d** Electron energy band diagram of PSC–TEG integrated device [64]. **e** Curves of current–voltage (I-V) and power output-voltage (P–V) obtained from PSM1 (6 series-connected sub-cells, active area 8.1 cm^2^) [62]. **f** Stability test of PSM1 and PSM1/TEG under continuous illumination [62]. **g** Schematic diagram of flat and concentrator photovoltaics with the architecture of glass/FTO/c-TiO_2_/m-TiO_2_/CsPbBr_3_/carbon [76]. **h** Schematic diagram for the concentrator CsPbBr_3_/Bi_2_Te_3_-integrated device [76]. **i** Schematic of the proposed SECS system consisting of a PSC integrated with a membrane-free TREC-RFB cell for concurrent solar energy storage, heat regulation, and waste heat harnessing [77]

Thermoelectric generator (TEG) is a solid-state device that converts thermal energy into electrical energy through the Seebeck effect. As shown in Fig. 5c, when a temperature gradient is applied across a semiconductor, charge carriers migrate from the hot side to the cold side, resulting in the accumulation of charge carriers and the formation of an electric potential difference [62, 63]. This potential difference drives a reverse charge flow until dynamic equilibrium is reached between the thermal motion and the internal electric field, establishing a stable thermoelectromotive force. The theoretical foundation of photovoltaic-thermoelectric (PV-TE)-integrated systems dates back to the 1970s [64]. Early research primarily focused on Si solar cells [65, 66] and other photovoltaic technologies [67–70]. In recent years, significant progress has been made in applying this strategy to PSCs. When integrated with PSCs, TEGs can effectively utilize waste heat to improve both efficiency and stability.

Theoretical estimation suggests that PSCs with an excellent Seebeck coefficient are more suitable for integration into PV-TE systems [71, 72]. Currently, many studies have adopted effective thermal management strategies by combining TEGs with carbon-based PSCs. On the one hand, the excellent near-infrared light absorption capability of carbon electrode can capture the light that perovskite layer cannot utilize. On the other hand, the superior photothermal conversion capability of carbon electrode provided a good hot end for TEG. Liu et al. demonstrated a novel integration of carbon-based PSCs and TEG [73]. As shown in Fig. 5a, solar radiation was mainly distributed in the spectral range of 300–1400 nm, with FTO and TiO_2_ exhibiting excellent transparency in this range. Perovskite exhibited excellent light capture capability from 300 to 800 nm. After directly printing carbon film on the perovskite layer, the absorption range was extended to 1400 nm, and the absorption capacity was further enhanced. They also recorded the temperatures of films under sunlight, both the perovskite film and the carbon film exhibited excellent photothermal conversion capabilities (Fig. 5b). If this portion of energy is not utilized properly, it can lead to a significant decrease in PSCs performance. As shown in Fig. 5c, by connecting the PSC and the TEG in series (carbon film and TEG hot end directly connected using thermal conductive silicone grease), the carbon film transferred the collected heat to TEG, forming a temperature gradient [73]. Based on the Seebeck effect, a voltage was obtained from the temperature difference between hot end and cold end, compensating for the voltage drop of PSC. Additionally, due to the Peltier effect, the conversion of thermal energy can lower the temperature of hot end. The efficiency and stability of PSCs based on thermoelectric integration were significantly improved, providing a new approach for thermal management.

Xu et al. also combined hole-conductor-free PSCs based on TiO_2_/ZrO_2_/carbon structures with TEG [64]. The band diagram is shown in Fig. 5d. The optimized integrated device achieved a maximum PCE of 20.3% with a *V*_oc_ of 1.29 V under 100 mW cm^−2^ illumination and demonstrated good operational stability. However, so far, PSC–TEG-integrated device has only been implemented in small areas, and manufacturing large-area PSC modules with variable series and parallel sub-cell configurations to match with the same size TEGs remains challenging. Fu et al. prepared large-area (16 cm^2^) PSCs and TEG-integrated device to prove the feasibility of large-area PSC–TEG-integrated device [62]. The integrated device achieved a maximum PCE of 12.7% and retained 85% of its initial PCE after continuous illumination for 400 h (Fig. 5e, f).

Concentrated photovoltaic (CPV) systems are often considered as a solution to surpass the Shockley–Queisser efficiency limit of single PN junction solar cells. When using CPV systems to increase the irradiance intensity beyond a standard solar level, the waste heat generated by solar cells also increases [74, 75]. Guo et al. cleverly combined TEG with CPV PSCs to fully utilize the entire solar spectrum (Fig. 5g, h). Apart from the excellent light utilization efficiency, the waste heat in PSC was also effectively harnessed and the integrated devices maintained over 90% of the initial PCE after 150 h irradiation under 5 suns, indicating excellent thermal stability [76]. This novel concept also reminds us to develop more optical design strategies to balance concentrated optics and heat utilization, promoting the advancement of cost-effective CPV–PSC–TEG technology in future. Interestingly, Zhang et al. proposed a technique to integrate PSC with a membrane-free zinc/manganese-based redox flow battery (RFB), which has a considerable negative temperature coefficient and low cost [77]. Since they also utilized the waste heat generated by PSCs and converted it into electrical energy, their research was also presented in this section. As shown in Fig. 5i, the PSC was fixed onto a custom graphite cooling plate. The electrolyte of RFB circulated through the cooling plate via serpentine channels, dissipating the heat generated by PSCs. Due to the efficient cooling effect, the PSC retained 96% of its initial PCE after operating at 35 ℃ for 70 h under one sun illumination.

Meanwhile, thermally regenerative electrochemical cycle allowed RFB to generate additional electricity from the low-grade heat gathered from PSC at a high absolute thermoelectric efficiency, thereby improving the overall system efficiency. Furthermore, RFB timely stored the electricity produced by PSC, buffering the fluctuation of solar power, stabilizing the electrical grids, and reducing the energy curtailment. This solar energy conversion and storage system, which featured concurrent solar energy storage, heat regulation, and waste heat harnessing, was of great significance for large-scale solar power generation [78–80].

To conclude, while PSC–TEG hybrid systems demonstrate remarkable potential, their practical deployment is hampered by notable challenges related to structural complexity and scalability. The integration demands stable thermal and electrical interfaces between the photovoltaic and thermoelectric parts, and it frequently relies on auxiliary cooling systems to sustain an effective temperature gradient. This not only complicates device design but may also impact long-term reliability. Moreover, upscaling these systems to the module or panel level brings about engineering hurdles, especially in guaranteeing uniform thermal contact and efficient heat transfer across large areas. Future studies ought to concentrate on simplifying the integration procedure, optimizing interface design, and evaluating the scalability and durability of these systems in real-world scenarios.

### Advanced Radiative Cooling for Efficient PSC Thermal Regulation

Radiative cooling is an effective strategy for passive cooling of PSCs with large surface areas facing the sky under outdoor conditions [81]. It utilizes the transparency of the Earth’s atmosphere to infrared radiation in the wavelength range of 8–14 µm, known as the atmospheric window, to achieve passive cooling [82]. Due to the existence of atmospheric window, the infrared radiation emitted by PSCs can escape into space without being absorbed by the Earth’s atmosphere, effectively dissipating heat and reducing the surface temperature of PSCs.

**Fig. 6:**
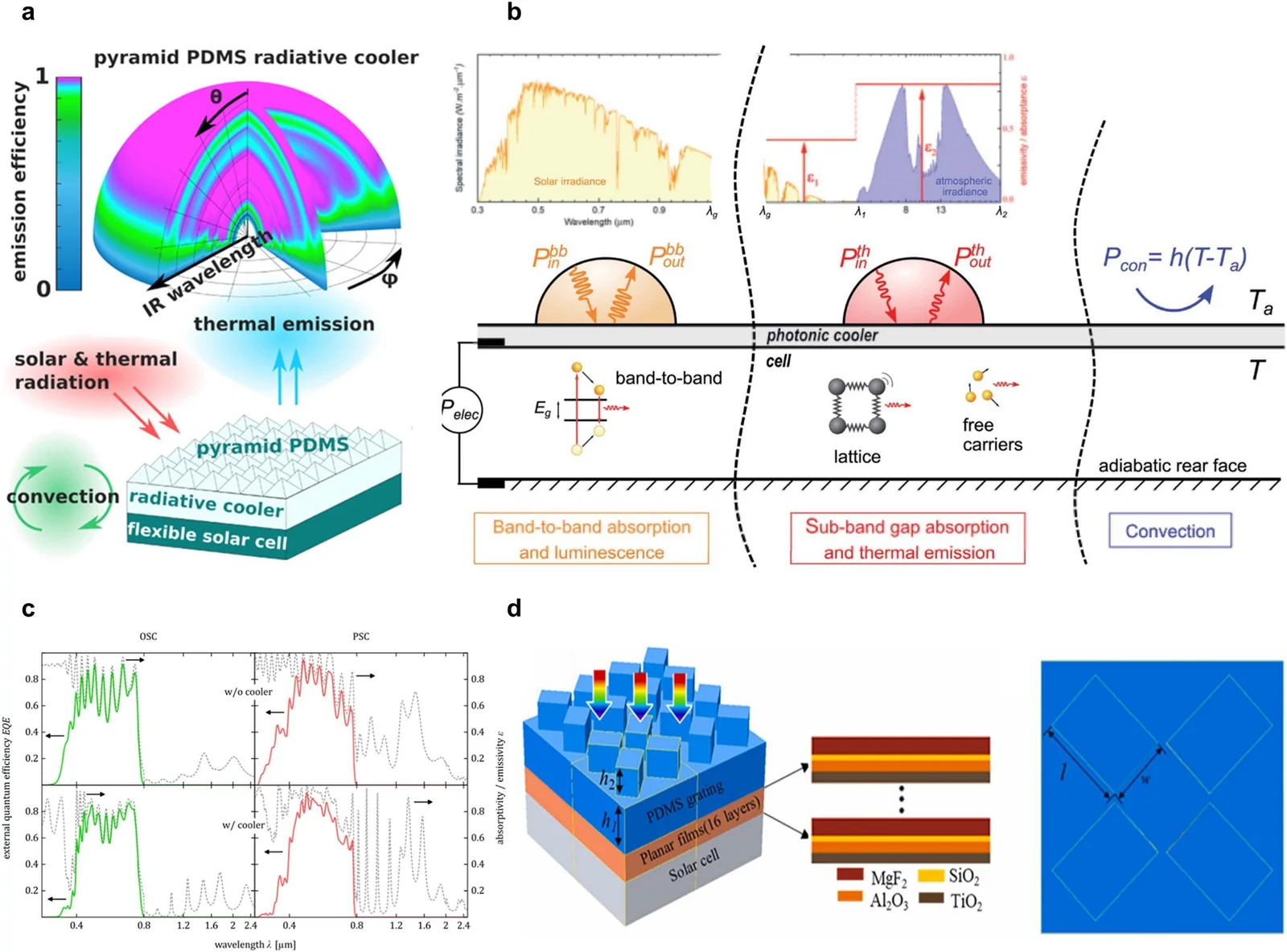
**a** Schematic diagram of solar cells with radiative cooling substrate and calculated emission efficiency contour of the radiative cooling substrates with pyramid-structured PDMS [83]. **b** Schematic illustration of the problem and model parameters [81]. **c** External quantum efficiency, and absorptivity/emissivity ε spectra (gray-dashed) at normal incidence of OSC (green-solid) and PSC (red-solid) without (top) and with a photonic cooler (bottom) [85]. **d** Structure of the cooling film. It includes multilayer film and PDMS grating layer. The multilayer stack is composed of MgF_2_, SiO_2_, Al_2_O_3_, and TiO_2_ [84]

Lee et al. conducted theoretical research on the use of polydimethylsiloxane (PDMS) as an effective thermal radiator for flexible thin-film solar cells [83]. Research has found that a 200 μm-thick planar PDMS layer can achieve a high emissivity of over 0.9 in the infrared range of 4–26 μm, and by introducing a pyramid structure on the PDMS, the emissivity can approach 1 in the range of 8–13 μm (Fig. 6a). The authors calculated the radiative cooling rate and found that the pyramid-structured PDMS layer can reduce the temperature of organic, perovskite, and microcrystalline Si flexible solar cells by 11, 12, and 16 ℃, respectively, compared to the commonly used polyethylene terephthalate (PET) substrate.

To gain a deeper understanding of physical mechanisms behind the cooling strategies, Dumoulin et al. proposed a comprehensive multidimensional and multiphysics OET modeling approach to study the radiative cooling systems of PSCs [81] (Fig. 6b). They designed a radiative cooler with near-ideal spectral selectivity, taking into account the coupling effects between radiative cooling characteristics, carrier thermodynamics, and electrodynamic behavior. The study results indicated that using such a photonic radiative cooler could lower the temperature of PSCs by more than 10 °C and increase the PCE by 0.45%. Building on this, Perrakis et al. proposed an ultra-thin, sub-micron-level organic–inorganic hybrid radiative cooler that was perfectly compatible with organic solar cells (OSCs) and PSCs [84]. This thermal management strategy can reduce the temperature by up to 17 K without any external energy input. It not only significantly lowered the PSC temperature but also improved the PCE while meeting the high-performance requirements of light weight, high stability, and flexibility (Fig. 6c).

Zhan et al. proposed a radiative cooler based on a grating-textured PDMS photonic structure that effectively reduced the operating temperature of PSCs and improved the PCE [85], as shown in Fig. 6d. The radiative cooler exhibited high average emissivity in the atmospheric transparency window, reflecting the solar radiation and radiating the waste heat into cold space. In addition, it had characteristics that were independent of angle and polarization, making it highly suitable for PSCs. Numerical analysis showed that this radiative cooler can ideally achieve a temperature reduction of 11.47 K for encapsulated PSCs.

Although PDMS-based radiative coolers offer a simple and effective means of passive heat dissipation, their long-term stability may be limited by UV-induced degradation under continuous outdoor exposure. To address this issue, recent studies have proposed the use of inorganic multilayer films as durable alternatives. Such designs, exemplified by SiO_2_/Si_3_N_4_ multilayer structures, can achieve high infrared emissivity and excellent UV resistance. Moreover, these multilayer radiative coolers can be directly integrated onto PSC surfaces by omitting the back-reflection layer, enabling efficient and stable thermal management without compromising optical performance [86].

In summary, as an advanced thermal management strategy for PSCs, the external integration of radiative coolers has successfully reduced PSC operating temperatures and enhanced overall performance through innovative designs of various materials and structures. It should be noted, however, that the practical implementation of radiative cooling also presents certain challenges, such as potential reflection losses caused by the cooler layer and compatibility issues with existing encapsulation materials, which may influence the overall optical efficiency and long-term stability of the device. Despite these considerations, current studies not only provide effective solutions for the thermal management of PSCs but also lay the foundation for the development of highly efficient and stable PSCs in future.

## Strategies for Reducing Heat Generation Within PSCs

While the previous section has focused on the effective dissipation of heat already generated within PSCs (e.g., through thermal conduction, radiative cooling, and thermoelectric conversion), an equally crucial approach involves suppressing heat generation at its source. In this section, we shift our focus to strategies that aim to reduce the intrinsic thermal effects in PSCs, primarily through down-conversion (DC) materials and tandem solar cell (TSC) structures. By minimizing energy losses that would otherwise be converted into heat, these methods complement the dissipation-focused strategies, together forming a comprehensive thermal management framework. The key parameters of the strategies discussed in this section are summarized in Table 2.

**Table 2:** Optoelectronic performance and stability of PSCs with different thermal management strategries to reduce heat generation

Thermal management strategies	*V*_oc_ (V)	*J*_sc_ (mA cm^−2^)	FF (%)	PCE (%)	Stability/thermal performance of device	Ref
Introducing NaYF_4_:Eu^3+^ nanophosphors down-conversion materials into PSCs	1.127	23.22	77.10	20.17	Retained 70% of its initial PCE after UV irradiation for 10 h	[97]
Introducing Sr_2_CeO_4_:Eu^3+^ nanophosphors down-conversion materials into PSCs	1.060	23.70	75.53	18.95	Retained 78% of its initial PCE after 75 days under ambient environment	[98]
Introducing Sr_2_CeO_4_:Sm^3+^ nanophosphor down-conversion materials into PSCs	1.050	23.65	72.40	17.90	Retained 50% of its initial PCE under ambient environment conditions for 60 days	[99]
Amorphous mesoporous TiO_2_ nanocrystals with surface-attached Eu^3+^ as a structural strategy for PSCs	1.080	19.34	75.67	15.79	–	[100]
Photocurable fluoropolymers for PSCs	1.090	23.23	74.00	18.67	Retained 95% of its initial PCE under ambient environment conditions for 92 days	[101]
DC layer of sinapoyl malate at the ETL/perovskite interface	–	–	–	20.50	Retained 95% of its initial PCE after aging at 100 °C in N_2_	[103]
Introducing CsPbCl_3_:Mn^2+^ quantum dots into PSCs	1.105	22.03	76.30	18.57	–	[104]
Stable low-bandgap Pb–Sn binary perovskites for TSCs	–	–	–	19.08	Retained 80% and 94% of its initial PCE after 12 and 30 days of storage in ambient and inert atmospheres	[111]
A universal close-space annealing strategy enabling efficient all-perovskite TSCs	1.956	15.41	83.10	25.05	Retained 90% of its initial PCE for about 450 h under continuous AM 1.5G solar illumination in a glovebox	[112]
Perovskite-perovskite tandem photovoltaics with optimized band gaps	1.660	14.50	70.00	16..90	–	[113]
Perovskite-organic TSCs	–	–	–	14.04	Retained 99.4% of its initial PCE after 120 h of strong UV irradiation at 100 mW cm^−2^	[116]
Perovskite-organic TSCs	2.150	14.00	80.00	24.00	More than 1000 h with no sign of degradation when the devices were kept under inert atmosphere, and a *T*_80_ of 130 h under continuous operation at the MPP	[120]
Solution-processed perovskite-colloidal quantum dot TSCs	–	–	–	20.55	The 4T tandem devices showed an MPP degradation with time, with the PCE decreasing to 90% of its initial PCE after 12.5 h	[124]

### Minimizing Thermalization Losses in PSCs with DC Materials

The DC process can be explained as the conversion of high-energy photons into two or more low-energy photons, also known as quantum clipping [87–89]. Integrating DC materials into PSCs is a feasible thermal management strategy because it can eliminate the spectral matching load of perovskites and minimize the energy used for thermalization heat, thereby reducing the generation of waste heat. To achieve a more efficient DC effect, researchers need to design and synthesize efficient DC materials which apply to PSCs. DC materials must have high emissivity, and light-absorption band should not overlap with the absorption range of perovskite [2]. Therefore, luminescent materials with a central absorption peak located in the UV region of solar spectrum can be selected as DC materials. DC materials can also effectively reduce the performance degradation of PSCs caused by high-energy UV light and recycle UV light for photocurrent generation in PSCs. Currently, many types of DC materials, including rare-earth-doped materials, semiconductor quantum dots, silicon nanocrystals, carbon-based materials, and metal complexes, exhibit excellent fluorescence properties [90–95]. Therefore, we primarily classify and summarize the thermal management strategy based on the types of DC materials, in order to facilitate readers’ understanding of progress and prospects of DC materials in PSCs thermal management.

**Fig. 7:**
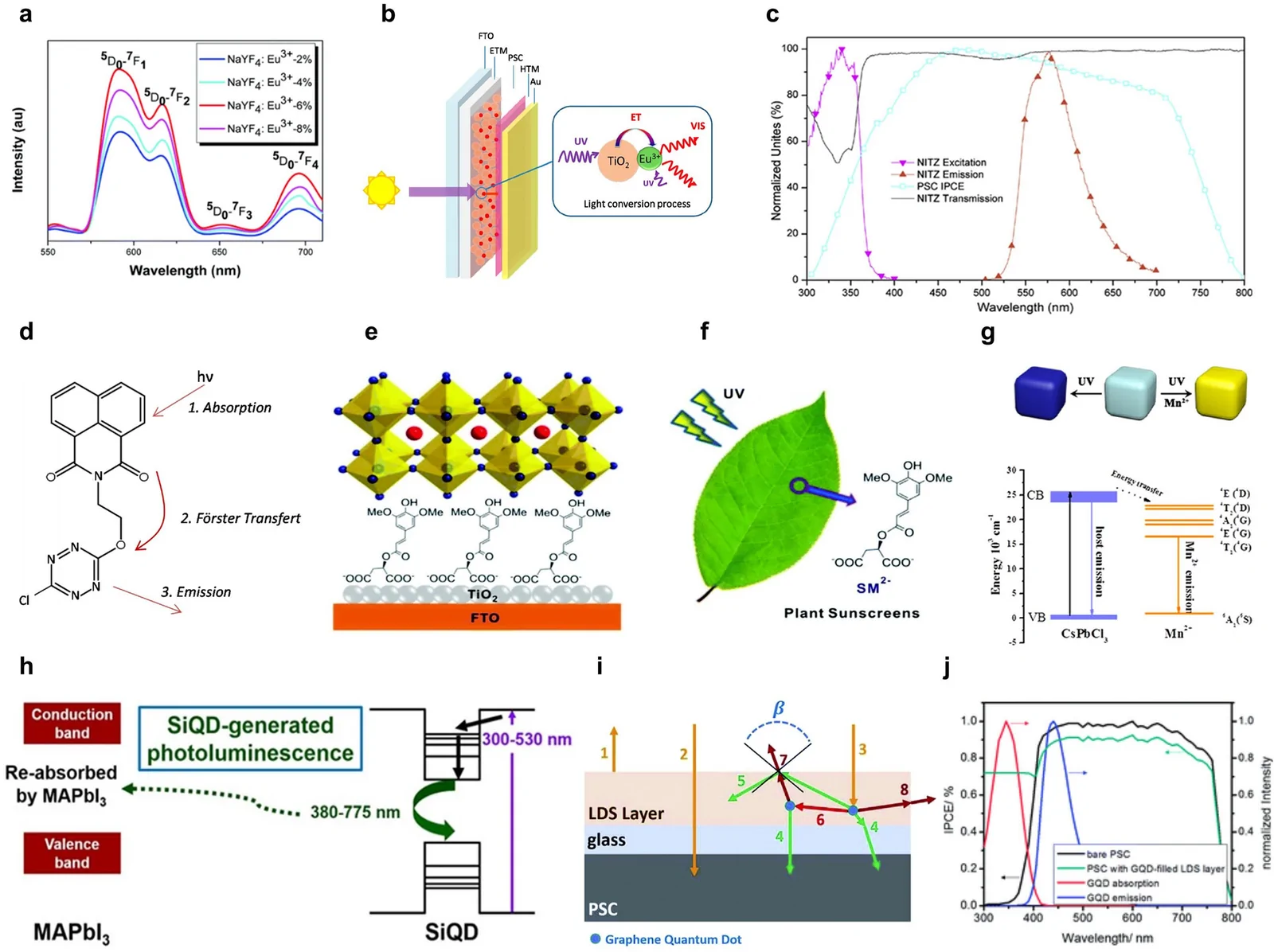
**a** Photoluminescence emission spectra of NaYF_4_:Eu^3+^ powder with an excited wavelength at 375 nm [97]. **b** Schematic diagram of the mechanism explaining the enhanced photoelectric performance of PSC with TiO_2_:Eu^3+^ [100]. **c** Excitation (magenta line) and emission (orange line) spectrum of NITZ molecule [102]. **d** NITZ molecule and associated photophysical steps [102]. **e** Schematic illustration of sinapoyl malate assembled at the interface between TiO_2_ and perovskite [103]. **f** Schematic illustration of the source and chemical structure of sinapoyl malate [103]. **g** Energy band gap alignment diagram of CsPbCl_3_:0.1Mn^2+^ quantum dots [104]. **h** Schematic diagram of quantum-assisted sunlight reabsorption processes at MAPbI_3_/Si quantum dot interface [105]. **i** Schematic diagram of optical processes involved in the graphene quantum dot-filled luminescent down shifting (LDS) layer on top of PSC [106]. **j** Normalized absorption and emission spectra of GQD, and IPCE spectra of PSCs without and with GQD-filled LDS layer [106]

Rare-earth-doped phosphors, also known as lanthanide-doped phosphors, are ideal candidates for DC materials. The electrons of lanthanide elements are individually filled in the 4f orbitals [96]. These elements can exist as trivalent cations and exhibit sharp and stable luminescence, with their emission positions unaffected by the surrounding matrix because they are protected by the outermost 5*s* and 5*p* orbitals. Due to the presence of the 4*f* subshell, energy levels are distributed diversely through interactions between ions, providing pathways for interband transitions. If an electron is in a highly excited state, relaxation processes may occur twice or more, which is the working principle of such DC materials. Jia et al. deposited NaYF_4_:Eu^3+^ on the non-conductive side of conductive glass to expand the spectral response range of PSCs. NaYF_4_:Eu^3+^absorbed UV light and exhibited emission peaks at 595, 614, 650, and 700 nm, corresponding to the typical 5D0–7F1, 5D0–7F2, 5D0–7F3, and 5D0–7F4 transitions of Eu^3+^ [97] (Fig. 7a). As a result, NaYF_4_:Eu^3+^-modified PSC achieved a PCE of 20.17% and a *J*_sc_ of 23.22 mA cm^−2^, higher than the control PSC’s 16.99% and 20.65 mA cm^−2^. In addition, NaYF_4_:Eu^3+^-modified PSC retained 70% of its initial PCE after UV irradiation for 10 h. Rahman et al. found that Eu^3+^-based DC nanomaterial Sr_2_CeO_4_:Eu^3+^ could also achieve a *J*_sc_ of 23.70 mA cm^−2^ and a PCE of 18.95% due to the collection and re-emission of UV light within the visible range via DC process [98]. They also reported that Sr_2_CeO_4_:Sm^3+^-modified PSC exhibited an enhancement of 11.4% in photocurrent and 16.2% in PCE [99]. PSCs based on Sr_2_CeO_4_:Eu^3+^ and Sr_2_CeO_4_:Sm^3+^ showed enhanced UV stability. PSC based on Sr_2_CeO_4_:Eu^3+^ retains 78% of its initial PCE after 75 days under ambient environment with RH = 20–25%. PSC based on Sr_2_CeO_4_:Sm^3+^ retains 50% of its initial PCE after 60 days of storage under ambient environment conditions. Additionally, Jiang et al. found that Eu^3+^ ions may adsorb onto the surface of TiO_2_ rather than substitute for Ti in the TiO_2_ lattice, attributed to the larger ionic size of Eu^3+^ compared to Ti^4+^ [100]. UV photons are captured by TiO_2_, and the energy is subsequently transferred to Eu^3+^, after which the DC process occurs (Fig. 7b).

In addition to traditional rare-earth-doped DC materials, organic materials with high fluorescence efficiency have also been developed to improve light utilization in PSCs. Bella et al. prepared a fluoropolymer (V570) coating on the surface of PSCs, which could enhance UV light utilization and photostability, significantly increasing incident photon-to-electron conversion efficiency (IPCE) [101]. At the same time, this coating can form a moisture barrier on the backside. In addition, PSC based on this strategy exhibited excellent operation stability, maintaining 95% of its initial PCE after 92 days of aging under ambient environment conditions. Gheno et al. introduced an S-tetrazole molecule (NITZ) containing polystyrene and N-(2-(6-chloro-tetrazol-3-oxyethyl)-naphthalimide) as a DC coating on a transparent conductive substrate of PSCs [102]. The excitation (magenta line) and emission (orange line) spectrum of NITZ molecule were shown in Fig. 7c. The molecular structure of NITZ is shown in the Fig. 7d, where the naphthalimide had a good absorption coefficient and transferred energy to the tetrazole via the Förster mechanism. NITZ as a DC layer exhibited good optical performance, showing light emission in visible region when excited by UV light. However, coatings outside PSC inevitably absorbed some light, which can lead to a decrease in PCE of PSCs. Therefore, Cao et al. introduced a DC layer of sinapoyl malate at the ETL/perovskite interface (Fig. 7e), inspired by natural compounds (Fig. 7f), significantly improving the PCE up to 20.5%. Furthermore, PSC based on this strategy retained 95% of its initial PCE after aging at 100 °C in N_2_ [103].

Recently, semiconductor quantum dots have been considered an excellent approach for achieving DC and spectral broadening in PSCs and other typical PV devices. Wang et al. found that doping Mn^2+^ into CsPbCl_3_ quantum dots could induce intermediate quantum states and increase quantum yield to around 60% [104] (Fig. 7g). Mn^2+^-doped CsPbCl_3_ quantum dots could enhance PCE and external quantum efficiency. Furthermore, the stability of PSCs has also been improved from 85% to 97% of their initial efficiency after exposure in the UV region with 5 mW cm^−2^ intensity by 100 h. The PCEs of organic and Si solar cells using Mn^2+^-doped CsPbCl_3_ quantum dots were increased by 3.21 and 2.98%, respectively. Besides, Si quantum dots are also very attractive in PV devices due to their excellent properties, including highly confined photoluminescence, non-toxicity, and abundant earth reserves. More importantly, Si quantum dots exhibit efficient DC effect, converting shorter wavelength excitation light (300–530 nm) into light in the visible region (550–800 nm). Wang et al. introduced Si quantum dots into PSCs to expand the external quantum efficiency over a wide wavelength range of 360–760 nm [105] (Fig. 7h). By converting short-wavelength (300–530 nm) light into visible light in the perovskite operating band (380–775 nm), Si quantum dots-modified PSC achieved an enhanced *J*_sc_ of 21.2 mA cm^−2^ and a PCE of 19.3%, along with improved thermal stability. These studies provided successful strategies for efficiently utilizing UV light and reducing thermalization losses of PSCs by introducing quantum dots.

Carbon materials are also considered good candidates for DC materials. Some typical and well-known carbon materials, particularly graphite, carbon black, carbon nanotubes, graphene, and graphyne, possess DC luminescence properties. From a theoretical simulation perspective, Hosseini et al. applied luminescent graphene quantum dots to the top of PSCs to improve light capture efficiency and stability [106]. Their calculations showed that a graphene quantum dots layer with a quantum yield of 94% could significantly increase IPCE, boosting photocurrent by more than 40% in the 300–400 nm spectral range (Fig. 7i, j).

From the above studies, we can conclude that DC materials play a key role in highly efficient PSCs by effectively utilizing UV light to reduce thermalization losses. This approach can alleviate the spectral matching load of PSCs and minimize thermal degradation caused by heat. In future, with the continuous optimization of DC materials and the combination with other thermal management methods, the photothermal stability and overall performance of PSCs are expected to be further enhanced, providing a broad prospect for achieving more efficient and stable PV devices.

### Minimizing Thermalization Losses in PSCs with Tandem Solar Cells

Research has shown that the excess energy generated by high-energy photons in PSCs is usually lost through thermalization [39]. TSCs can maximize light utilization across the spectral response range. Typically, TSCs consist of two or more sub-cells with complementary absorption spectra, featuring a wide bandgap (WBG) and a narrow bandgap (NBG). The WBG sub-cell absorbs high-energy photons, while the NBG sub-cell absorbs low-energy photons, thereby minimizing thermalization losses and managing the thermal effects in PSCs [6, 107–109].

TSCs are primarily divided into two types [110]. The first type is the 4-terminal (4T) structure, where the two cells are independent and not electrically connected. In a 4T structure, the top cell must be semitransparent to allow light to pass through and be absorbed by the bottom cell [2]. The second type is the 2-terminal (2T) structure, which requires more complex manufacturing processes [10]. In a 2T structure, the *V*_oc_ is the sum of the *V*_oc_ of two sub-cells. However, the* J*_sc_ is limited by the lower *J*_sc_ of sub-cells. Therefore, current matching is crucial for 2T TSCs, meaning that the bandgaps and thicknesses of the two absorber layers must be precisely matched. Compared to the 4T structure, the 2T structure can minimize parasitic absorption losses and improve practical efficiency.

**Fig. 8:**
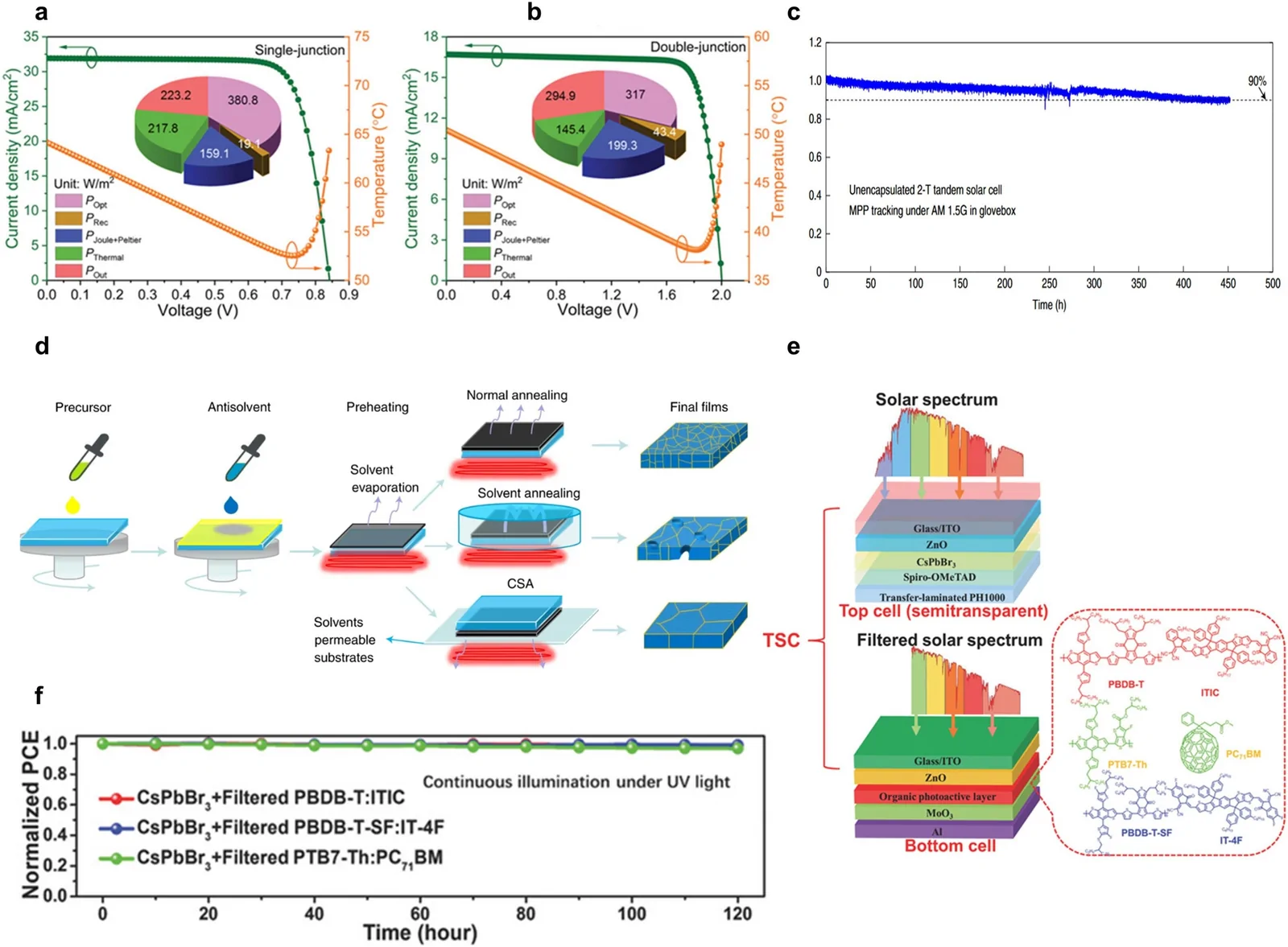
*J*–*V* curves and temperature plots under the various biases of the **a** single- and **b** double-junction tandem PSCs [36]. The microscopic energy conversion components at MPP are inserted in the figure. Here, *P*_Opt_, *P*_Rec_, *P*_Joule+Peltier_, *P*_Thermal_, and *P*_Out_ denote the power densities for optical loss, recombination, intrinsic loss (including Joule and Peltier losses), thermalization, and electrical output processes, respectively. **c** MPP tracking of one unencapsulated 2T all-perovskite TSC under continuous AM 1.5 g 1 sun illumination in a glovebox; the initial efficiency is 24.5% [112]. **d** Schematic diagram of normal annealing, solvent annealing and CSA processes [112]. **e** Device structure of four-terminal TSCs: semitransparent top cell with a structure of glass/ITO/ZnO/CsPbBr_3_/Spiro-OMeTAD/transfer-laminated PH1000; bottom OSCs with a structure of glass/ITO/ZnO/organic photoactive layer/MoO_3_/Al, and molecular structures of donor and acceptor materials used in the corresponding organic photoactive layer [116]. **f** Normalized PCEs *versus* storage time under UV light (365 nm, 100 mW cm^−2^) illumination in a N_2_-filled glovebox [116]

First of all, all-perovskite tandem solar cell utilizes a WBG PSC as the top cell and a NBG PSC as the bottom cell. For the 4T structure, each sub-cell can be optimized independently during fabrication, without constraints. Ma et al. developed an OET model to simulate the suppression of thermodynamic losses in multi-junction solar cells, providing a microscopic quantitative analysis to reveal the inherent thermodynamic behavior of multi-junction tandem solar cells [36]. Figure 8a, b presents the *J*–*V* curves, temperature curves, and energy distribution of single-junction and double-junction TSCs at MPP. The results indicate the following: (1) Due to more efficient light absorption, thermalization losses significantly decrease from 217.8 W m^−2^ (single-junction) to 145.4 W m^−2^ (double-junction), confirming the feasibility of tandem design in reducing thermalization losses. (2) The predicted temperature at MPP for the double-junction device is 37.9 °C, much lower than the 51.8 °C observed in the single-junction device, due to the lower heat generation and higher energy utilization. Yang et al. achieved a 19.8% PCE using a bottom cell Sn-based PSC with a bandgap of 1.33 eV and a top cell based on MAPbI_3_ [111]. The TSC based on this strategy retained 80% and 94% of its initial PCE after 12 and 30 days of storage in ambient and inert atmospheres, respectively. Wang et al. employed (FASnI_3_)_0.6_(MAPbI_3_)_0.4_ (bandgap of 1.25 eV) and FA_0.8_Cs_0.2_Pb(I_0.7_Br_0.3_)_3_ (bandgap of 1.75 eV) to fabricate tandem solar cells, achieving an efficiency of 25.15% by using a universal close-space annealing (CSA) strategy to increase grain size, enhance crystallinity, and prolong carrier lifetime in both narrow and wide bandgap perovskite layers [112] (Fig. 8d). The unencapsulated TSC presented excellent stability that retained 90% of its original PCE for about 450 h under continuous AM 1.5 G 1 sun illumination in a glovebox (Fig. 8c). For the 2T structure, in 2016, Eperon et al. first combined a perovskite with a bandgap of 1.2 eV (FA_0.83_Cs_0.25_Sn_0.5_Pb_0.5_I_3_) with a wider bandgap FA_0.83_Cs_0.17_Pb(I_0.5_Br)_3_, achieving a PCE of 16.9% and the *V*_oc_ exceeding 1.66 V [113]. All-perovskite TSCs were prone to the erosion of sub-cell solution during the deposition of the top perovskite layer, which increased the difficulty of the fabrication process [41].

OSCs were advantageous candidates for the bottom sub-cell of TSCs, as organic materials exhibited excellent spectral response in the near-infrared region and demonstrated higher stability compared to tin-based perovskite layers. Additionally, the use of intrinsic orthogonal solvent methods to dissolve organic–inorganic perovskite and organic materials makes them more competitive [114]. As a result, perovskite/organic TSCs have been extensively studied. Organic materials are prone to oxidation and degradation under UV radiation [115]. The WBG perovskite on ITO or FTO is advantageous for UV shielding. Chen et al. prepared a 4T perovskite/organic TSC with a structure consisting of a semitransparent perovskite top sub-cell (ITO/ZnO/CsPbBr_3_/Spiro-OMeTAD/PH1000) and an organic bottom sub-cell (ITO/ZnO/PBDB-T-SF:IT-4F/MoO_3_/Al) [116] (Fig. 8e). They used a dual-source vacuum evaporation (DSVE) method to prepare high quality CsPbBr_3_. This semitransparent perovskite not only exhibited an average visible light transmittance of about 90% in the 530–800 nm but also filtered out harmful UV light for the bottom cell, utilizing UV light for PV conversion. The 4T TSC based on PBDB-T-SF:IT-4F bottom PSC showed the best PCE of 14.03% and retained 99.4% of its initial PCE after 120 h of strong UV irradiation at 100 mW cm^−2^, demonstrating good stability (Fig. 8f). The results indicate that this perovskite/organic TSC not only reduces thermalization losses of unused higher-energy UV light but also improves the overall efficiency and stability of the device. For 2T perovskite/organic TSCs, the performance is relatively lower compared to all-perovskite TSCs and perovskite/Si TSCs due to the lower* J*_sc_ of the bottom organic sub-cell and the losses introduced by sub-cell interconnection [117, 118]. However, the emergence of narrow-bandgap non-fullerene acceptors has led to a rapid increase in the PCE of OSCs [119]. Brinkmann et al. demonstrated a perovskite/organic TSC with an efficiency of 24.0% and a high *V*_oc_ of 2.15 V [120]. Their work used an ultra-thin (1.5 nm) metallic indium oxide layer for connection, exhibiting unprecedentedly low optical/electrical losses. The device showed an excellent stability of more than 1000 h with no sign of degradation when the device was kept under inert atmosphere, and a *T*_80_ of 130 h under continuous operation at the MPP. This work sets a milestone for perovskite/organic TSCs. In addition to organic materials, quantum dots with low bandgaps can provide more complementary absorption in the near-infrared region and reduce energy losses. PbS quantum dots, due to the excellent advantages such as low cost, ease of processing, and tunable bandgap, are expected to become strong competitors for the bottom sub-cell of TSCs [121–123]. Importantly, controlling the size of PbS quantum dots can effectively adjust the bandgap to achieve complementary absorption.

Manekkatthodi et al. developed a 4T perovskite/PbS quantum dot TSC with a PCE exceeding 20% [124]. In this TSC, the front transparent PSC used a dielectric-metal-dielectric (DMD) electrode, which consisted of a metal film (silver/gold) sandwiched between dielectric (MoO_3_) layers, with the best PCE reaching 20.55%. Recently, in-depth research has also been conducted on 2T TSCs based on perovskite (*E*_g_ = 1.55 eV)/PbS quantum dots (*E*_g_ = 1.0 eV). Karani et al. designed a perovskite/PbS quantum dots TSC with a theoretical PCE of 43% under standard AM1.5G solar illumination [125]. The radiative coupling effect between the sub-cells can recycle emitted photons, significantly improving overall efficiency.

## Conclusions and Perspectives

In this review, we analyzed the degradation mechanisms of PSCs under high-temperature conditions, investigated the internal heat sources, and provided a detailed discussion of existing thermal management strategies. Thermal instability remains one of the major barriers to the commercialization of PSCs, underscoring the importance of effective thermal management. However, current research in this field remains limited, requiring deeper exploration and multidisciplinary innovation.

**Fig. 9:**
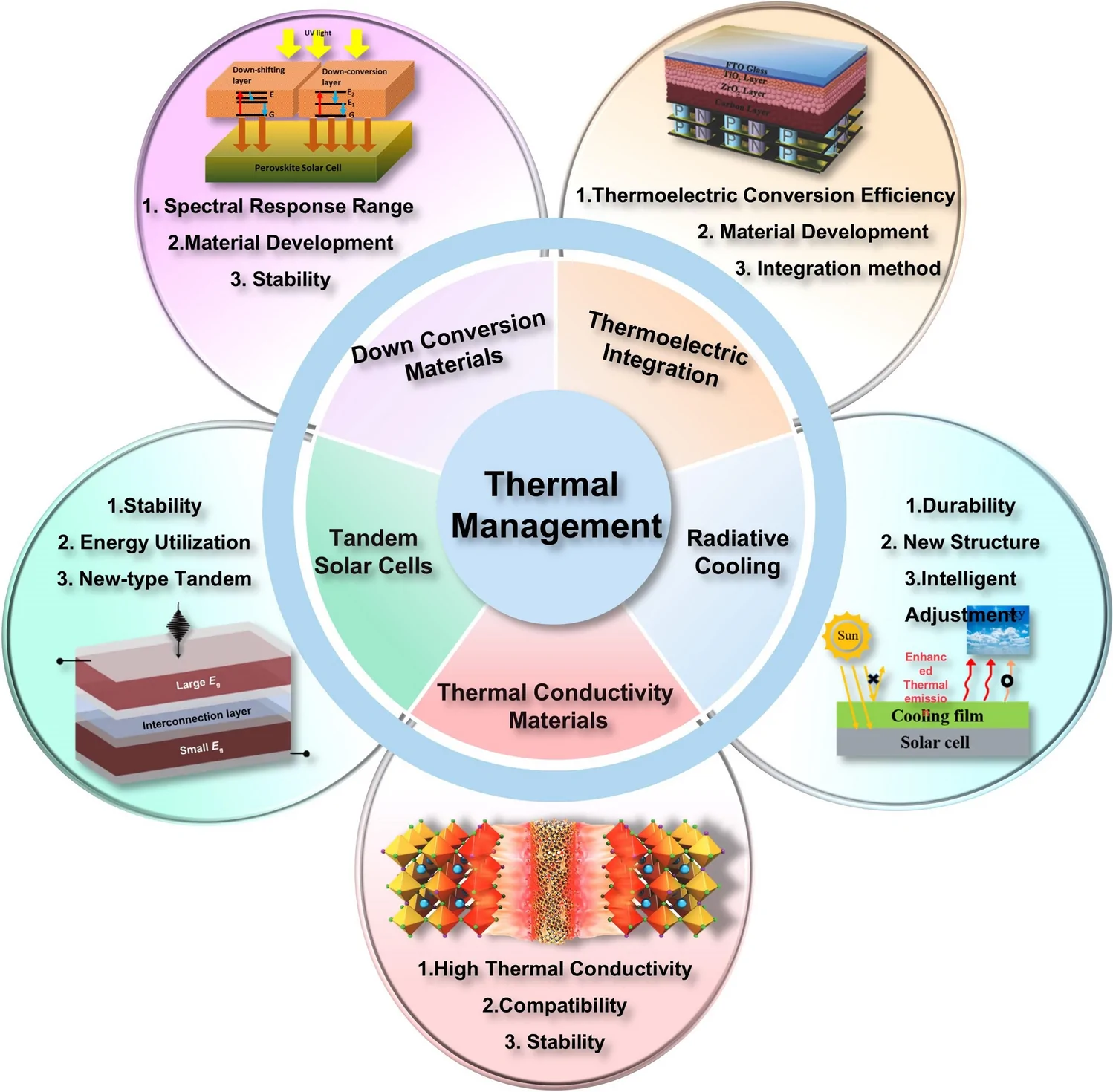
Schematic diagram of thermal management strategies in PSCs, covering advanced thermal conductive materials [51], radiative cooling designs [84], thermoelectric integration [62], DC materials [87], and tandem architectures [126]

Looking ahead, breakthroughs in PSC thermal management are expected to emerge from several key areas (Fig. 9). First, optimizing thermally conductive materials within PSCs will be essential for enhancing heat dissipation efficiency. Future efforts should focus on developing materials with high thermal conductivity, chemical stability, and interfacial compatibility to minimize thermal stress and defect formation, thereby extending device lifetime. Second, advanced radiative cooling designs will continue to evolve, offering passive and energy-free routes for temperature regulation. The development of durable and spectrally selective cooling layers, as well as the integration of adaptive temperature regulation mechanisms, will be crucial for maintaining stable operation without compromising optoelectronic performance. Third, hybrid photonic-thermoelectric systems represent a promising direction for coupling light management with waste heat recovery, enabling synergistic improvement in both energy utilization and stability. In addition, interface engineering and encapsulation technologies will play vital roles in mitigating thermal stress and improving long-term reliability, ensuring compatibility between thermal management layers and perovskite absorbers.

In summary, future progress in PSC thermal management will rely on the multidisciplinary integration of materials science, device engineering, and photonic design. Continued innovations in thermally conductive materials, radiative cooling architectures, thermoelectric integration, and interface optimization will provide powerful tools to improve both efficiency and durability, ultimately accelerating the commercialization of perovskite solar cells. In addition, the machine learning (ML) techniques offer promising opportunities for advancing PSC thermal management. ML algorithms can accelerate materials discovery for thermally conductive or radiative layers, optimize device architectures for improved heat dissipation, and enable predictive modeling of thermal degradation under diverse operating conditions. Incorporating data-driven approaches could therefore significantly enhance design efficiency and accelerate innovation in this emerging field.
